# Metabolic Plasticity of Glioblastoma Cells in Response to DHODH Inhibitor BAY2402234 Treatment

**DOI:** 10.3390/metabo14080413

**Published:** 2024-07-27

**Authors:** Ayenachew Bezawork-Geleta, Diane Moujalled, David P. De Souza, Vinod K. Narayana, James Dimou, Rodney Luwor, Matthew J. Watt

**Affiliations:** 1 Department of Anatomy and Physiology, School of Biomedical Sciences, The University of Melbourne, Melbourne, VIC 3010, Australia; 2 Blood Cells & Blood Cancer Division, The Walter and Eliza Hall Institute of Medical Research, Melbourne, VIC 3052, Australia; 3 Department of Medical Biology, The University of Melbourne, Parkville, VIC 3010, Australia; 4 Metabolomics Australia, Bio21 Institute, The University of Melbourne, Melbourne, VIC 3010, Australia; 5 Department of Surgery, The University of Melbourne, Parkville, VIC 3010, Australia; 6 Department of Neurosurgery, The Royal Melbourne Hospital, Parkville, VIC 3050, Australia; 7 Fiona Elsey Cancer Research Institute, Ballarat, VIC 3350, Australia; 8 Federation University, Ballarat, VIC 3350, Australia; 9 Huagene Institute, Kecheng Science and Technology Park, Pukou District, Nanjing 211806, China

**Keywords:** lipid metabolism, lipid droplets, brain cancer

## Abstract

Glioblastoma (IDH-wildtype) represents a formidable challenge in oncology, lacking effective chemotherapeutic or biological interventions. The metabolic reprogramming of cancer cells is a hallmark of tumor progression and drug resistance, yet the role of metabolic reprogramming in glioblastoma during drug treatment remains poorly understood. The dihydroorotate dehydrogenase (DHODH) inhibitor BAY2402234 is a blood–brain barrier penetrant drug showing efficiency in in vivo models of many brain cancers. In this study, we investigated the effect of BAY2402234 in regulating the metabolic phenotype of EGFRWT and EGFRvIII patient-derived glioblastoma cell lines. Our findings reveal the selective cytotoxicity of BAY2402234 toward EGFRWT glioblastoma subtypes with minimal effect on EGFRvIII patient cells. At sublethal doses, BAY2402234 induces triglyceride synthesis at the expense of membrane lipid synthesis and fatty acid oxidation in EGFRWT glioblastoma cells, while these effects are not observed in EGFRvIII glioblastoma cells. Furthermore, BAY2402234 reduced the abundance of signaling lipid species in EGFRWT glioblastoma. This study elucidates genetic mutation-specific metabolic plasticity and efficacy in glioblastoma cells in response to drug treatment, offering insights into therapeutic avenues for precision medicine approaches.

## 1. Introduction

Glioblastoma, isocitrate dehydrogenase (IDH)-wildtype, stands as the most prevalent form of primary adult brain cancer and continues to present a formidable clinical challenge [1]. The current standard of care for glioblastoma involves surgical resection of the tumor followed by a combination of radiation therapy and temozolomide (TMZ) chemotherapy. Although 80% of treated patients are responsive to such standard care of management within 6 months, the long-term prognosis remains bleak with only about 10% maintaining responsiveness at 24 months [2,3,4,5]. The complexity of glioblastoma heterogeneity, stemming from genetic mutations, epigenetic dysregulation, developmental factors, and the tumor microenvironment, underscores the multifaceted nature of therapeutic resistance and failure [6,7,8,9].

Metabolic vulnerability represents a phenotypic trait wherein cancer cells become reliant on particular metabolic pathways to sustain their rapid proliferation, survival, and adaptation to therapeutic interventions such as chemotherapy and radiotherapy [10]. A classic example of metabolic vulnerability in cancer is the Warburg effect [11], which delineates cancer cells’ preference for aerobic glycolysis to generate energy from glucose. This metabolic adaptation provides cancer cells with a growth advantage despite its inefficiency. Recent studies have shed light on cancer cells’ dependence on specific nutrients or metabolites for their growth and survival [10,12] with certain cancer types exhibiting heightened requirements for amino acids [13,14,15,16] or lipids [16,17,18,19,20,21] to fuel their accelerated growth. Inhibiting the uptake or metabolic processing of these essential macromolecules holds potential for limiting tumor progression. While exploiting these vulnerabilities holds promise for the development of targeted and effective cancer treatments, the intricate and interconnected nature of metabolic networks, coupled with their plasticity toward intrinsic and environmental stimuli, presents a challenge for cancer therapeutics [22]. 

Pyrimidine synthesis has been a key drug target for cancer therapeutics since the 1950s. Five-fluorouracil (5-FU), an inhibitor of thymidylate synthase, was an early drug for several cancer treatments [23,24,25] and remains widely utilized in laboratory investigations to explore cancer cell adaptation mechanisms to drugs. Another long-standing anticancer agent targeting pyrimidine synthesis is cytarabine [26].

Dihydroorotate dehydrogenase (DHODH) is a central enzyme of pyrimidine biosynthesis and catalyzes the oxidation of dihydroorotate to orotate. More recently, the dihydroorotate dehydrogenase (DHODH) inhibitor BAY2402234 showed blood–brain barrier penetration and promising efficacy in IDH mutant glioma, diffuse midline glioma, and MYC-amplified medulloblastoma. While two weeks of daily BAY2402234 administration is well tolerated in mouse models of diffuse midline glioma [27,28,29], long-term treatment is associated with relapse, suggesting potential metabolic adaptations in cancer cells.

Drug response in cancer patients is highly influenced by the genetic makeup of both the patient and the tumor. It has been shown that about 54% of glioblastoma patients express the EGFR WT protein, while 31% of patients express EGFRvIII [30]. We sought to examine the effects of BAY2402234 on cell viability and metabolic responses in patient-derived glioblastoma cells that differ in their EGFR gene. We show that BAY2402234 selectively kills glioblastoma cells expressing the EGFRWT protein but has minimal effects on cell viability in EGFRvIII-expressing glioblastoma cells. The effects of BAY2402234 were associated with the EGFR-dependent remodeling of lipid metabolism. This study provides the important understanding of metabolic alterations and drug resistance which has substantial implications for further clinical investigations leading to the development of novel multi-targeted inhibitors.

## 2. Materials and Methods

### 2.1. Cell Culture

GBM cell lines #35 and #41 which were originally derived from 2 patients with pathologically confirmed GBM at the Royal Melbourne Hospital were previously molecularly characterized and described previously [31]. Cells were cultured in high-glucose and GlutaMAX DMEM (Thermo Fisher Scientific; #11965092, Waltham, MA, USA) supplemented with 10% fetal bovine serum (FBS; Cell Sera; AU-FBS/PG, Rutherford, Australia) and 1% penicillin–streptomycin (10,000 U/mL; Thermo Fisher Scientific; 15140122, Waltham, MA, USA) in a humidified atmosphere of 5% CO_2_ at 37 °C. Cells tested negative for mycoplasma contamination using DAPI staining.

### 2.2. Cell Viability Assay

Cell viability assays were conducted in a white, flat-bottom, 12-well tissue culture plate (Corning, Corning, NY, USA). GBM cells (passage 5 to 10) were seeded at a density of 2.0 × 10^5^ cells/well 24 h prior to incubation with the inhibitor. Varying concentrations of BAY2402234 (or DMSO as control) were then added to each well, and the plates were incubated at 37 °C with 5% CO_2_. The medium was replenished with DMEM containing BAY2402234 daily. After treatment for 72 h, cells were incubated with MTT-labeling reagents for 4 h. Subsequently, the absorbance values at 570 nm and 660 nm were simultaneously measured using a Synergy™ Neo spectrofluorometer at 25 °C. 

### 2.3. Cell Lysis, SDS PAGE and Western Blot Analysis

Cells were incubated with 0, 5 and 10 nm Bay inhibitors for 48 h. The cells were lysed in RIPA lysis buffer (50 mM Tris HCL, pH 7.4; 150 mM NaCl; 0.1% SDS; 0.5% sodium deoxycholate; 1% Triton X-100) supplemented with protease inhibitor cocktail (cOmplete, Roche, Basel, Switzerland) and phosphatase inhibitor (PhosSTOP, Roche, Basel, Switzerland) on ice, and the cell debris was removed by centrifugation. Proteins were separated by SDS-PAGE. Proteins were then transferred to a nitrocellulose membrane (0.45 µm, #1620264; Bio-Rad, Hercules, CA, USA) followed by blocking with 5% (wt/vol) skim milk in TBS-T for 2 h at RT; then, they were immunoblotted with antibodies for caspase-3 (Cell Signaling Technology, #9662, Danvers, MA, USA) 1:1000, AKT (Cell Signaling Technology, #9272) 1:1000, MAPK (Cell Signaling Technology#9102 1:1000, Danvers, MA, USA), P53 (#DO-1, sc-126 Santa Cruz 1:1000, Dallas, TX, USA), β-actin (Santa Cruz sc-517582 HRP 1:5000, Dallas, TX, USA), and GAPDH (Thermo Fisher, #MA5-32539, Waltham, MA, USA). Incubation with primary antibody was performed overnight at 4 °C followed by three washes for 5 min each in TBS-T. After the unbound antibodies were removed, the membranes were incubated with the horseradish peroxidase secondary antibody (1:5000 dilution) for 1 h at room temperature. The bound antibodies were detected by Clarity Western ECL Reagent (Bio-Rad, Hercules, CA, USA) and visualized with Molecular Imager^®^ ChemiDoc™ XRS+ (Bio-Rad, Hercules, CA, USA). The chemiluminescence intensity of protein bands was analyzed and documented by Image Lab software (ver. 6.10 build 7, Bio-Rad, Hercules, CA, USA).

### 2.4. Metabolic Flux Analysis Using ^13^C-Fatty Acid Mix by Mass Spectrometry

GBM#35 and GBM#41 were preincubated for 48 h with high-glucose and GlutaMAX DMEM supplemented with 10% fetal bovine serum with 5 nM BAY2402234. Cells were then switched to serum-free low-glucose (5 mM) DMEM in the presence of 400 μM uniformly labeled ^13^C fatty acid mix (Cambridge Isotope Laboratories, Tewksbury, MA, USA) conjugated with 1% BSA and 5 nM BAY2402234 for 4 h. Cells were washed briefly with PBS and LC/MS-grade water before quenching with liquid nitrogen. 

Metabolites were extracted on ice by the addition of 600 µL/well of methanol:chloroform (9:1 *v*/*v*), containing the internal standard, scyllo-inositol (16.6 µM). Cells were scraped and incubated on ice for 10 min. Samples were then centrifuged (5 min, 14,000 rpm, 4 °C) to pellet precipitated proteins, and the supernatants were transferred to fresh Eppendorf tubes.

For analysis of stable isotope incorporation, cell extracts were transferred to vial inserts and evaporated to dryness under vacuum and then derivatized online using a Shimadzu AOC6000 autosampler robot (Shimadzu, Kyoto, Japan). Derivatization was achieved via the addition of 25 µL methoxyamine hydrochloride (30 mg/mL) in pyridine followed by shaking at 37 °C for 2 h. Samples were then silylated with 25 µL of N,O-bis (trimethylsilyl)trifluoroacetamide (BSTFA) with 1% trimethylchlorosilane (TMCS) for 1 h at 37 °C. Samples were allowed to equilibrate at room temperature for 1 h before 1 µL was injected onto the gas chromatography (GC) column using a hot needle technique. Split (1:10) injections were performed for each sample. The used GC-MS system comprised an AOC6000 autosampler and 2030 Shimadzu gas chromatograph coupled to a TQ8050NX triple quadrupole mass spectrometer (Shimadzu, Japan). The mass spectrometer was tuned according to the manufacturer’s recommendations using tris-(perfluorobutyl)-amine (CF43). GC-MS analysis was performed on a 30 m Agilent DB-5 column with a 0.25 mm internal diameter column and 1 µm film thickness. The injection temperature (inlet) was set at 280 °C, the MS transfer line was set at 280 °C, and the ion source was adjusted to 200 °C. Helium was used as the carrier gas at a flow rate of 1 mL/min. The analysis of derivatized samples was performed under the following oven temperature program: 100 °C start temperature, hold for 4 min, followed by a 10 °C min^−1^ oven temperature ramp to 320 °C with a following final hold for 11 min. The mass spectrometer was operated in electron ionization mode with a scan range of 45–600 *m*/*z* at a 2000 scan speed.

The semi-targeted central carbon metabolites and their mass isotopologues were integrated in the DExSI software (version 3.5) [32]. Each peak integration was visually validated and manually corrected where required. The DExSI output for each compound was the fractional labeling value of the total compound pool corrected for the natural isotopic background abundance.

### 2.5. Targeted Lipidomic Analysis

GBM#35 and GBM#41 were preincubated for 48 h with high-glucose and GlutaMAX DMEM supplemented with 10% fetal bovine serum with 5 nM BAY2402234 in a 6-well plate. Cells were then washed briefly with PBS and LC/MS-grade water before quenching with liquid nitrogen. For lipid extraction, 600 µL of methanol:chloroform (9:1) containing 10 mg/L of each internal standard was added to the 6-well plate. The cells were scraped with a cell lifter. The internal standards were PC19:0/19:0 (part 850367), PE-d31 (part 860374), PG17:0/17:0 (part 830456) and TG-d5 19:0/12:0/19:0 (Avanti, #8609040). Extracted solvent with cells was transferred into 2 mL fresh LoBind Eppendorf tubes, and 1 mL chloroform was added to each tube to bring the ratio of chloroform:methanol to 2:1. Samples were vortexed and then mixed at 950 rpm for 30 min at 20 °C with a Thermomixer (Eppendorf, Hamburg, Germany). Samples were centrifuged at 15,000 rpm (Beckman Coulter Microfuge^®^ 22R Refrigerated Microcentrifuge, Brea, CA, USA) for 10 min, and the supernatant was transferred to fresh LoBind Eppendorf tubes. Samples were completely dried in a vacuum concentrator with the temperature maintained at 30–35 °C (Christ^®^ RVC 2–33, Martin Christ Gefriertrocknungsanlagen, Osterode am Harz, Germany). The samples were reconstituted with water-saturated butanol:methanol (100 µL, 9:1, *v*/*v*). 

Pooled biological quality control samples (PBQCs) were prepared by pooling aliquots of the extracts from each sample and were run after every five samples. Extracted lipids were processed and detected by Metabolomics Australia (Bio21 Institute, Melbourne, VIC, Australia) as previously described [33,34] using an Agilent 1290 liquid chromatography (LC) system and Triple Quadrupole 6490 mass spectrometer (MS, Agilent Technologies Australia, Mulgrave, Australia). For LCMS analysis, nonpolar (lipid) extracts were analyzed by LCMS in positive ionization mode to obtain the most comprehensive coverage with dynamic scheduled multiple reaction monitoring. The MS parameters and MRM transitions of each lipid class, subclass and individual lipid species have been previously described [33,34]. Data processing was performed using Agilent’s Mass Hunter Quantitative Analysis (QQQ) software 10.0 (Agilent Technologies). Lipid species were named according to the LIPID MAPS nomenclature described previously [33].

## 3. Results

### 3.1. Pharmacological Inhibition of DHODH Selectively Kills GBM Subtypes

**Figure 1:**
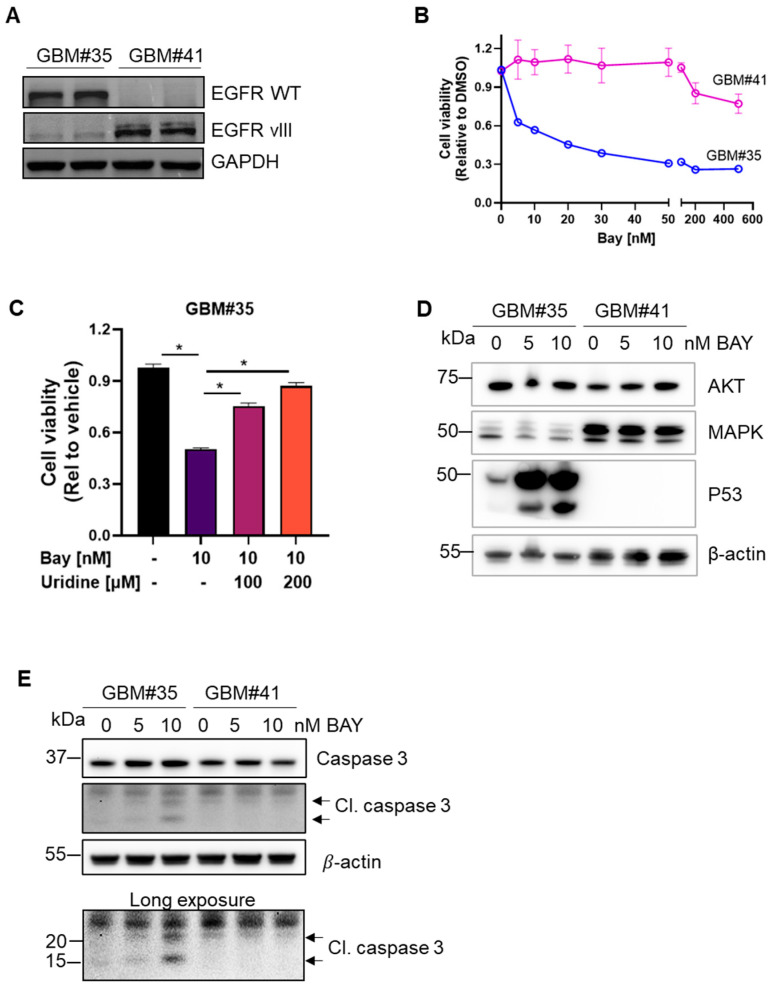
DHODH inhibitor BAY2402234 (Bay) selectively induced cell death in GBM subtype. (**A**) Western blot analysis of EGFRWT and EGFRvIII expression in patient-derived GBM#35 and GBM#41 cell lines. (**B**) Cell viability assay using MTT of GBM#35 and GBM#41 cell lines treated with Bay at indicated concentration for 72 h (n = 6, mean ± SEM). (**C**) Cell viability assay using MTT of GBM#35 cell lines treated with Bay in the presence of uridine at indicated concentration for 72 h (n = 8, mean ± SEM). Statistical significance was determined using one-way ANOVA with Bonferroni’s multiple comparison test, * *p*  <  0.05 vs. Bay treated (without uridine supplement). (**D**) Key oncogenic proteins expression in Bay-treated GBM#35 and GBM#41 cell lines. β-actin was used as loading control. (**E**) Bay dose-dependent cleavage of caspase 3 in GBM#35 as compared to GBM#41 cell lines. β-actin was used as loading control.

To assess the functional significance of EGFRWT and EGFRvIII in drug response, we utilized two patient-derived GBM cell lines originally obtained from patients with pathologically confirmed GBM and subsequently adapted from non-adherent neurosphere cells to adherent cells grown in monolayer culture [31]. Western blot analysis confirmed EGFR expression using antibodies specific to EGFRWT and EGFRvIII (Figure 1A). 

We assessed the efficacy of the DHODH inhibitor BAY2402234 against GBM cells expressing WT or mutant EGFR. The patient-derived cell line GBM#35 (EGFRWT) exhibited sensitivity to BAY2402234 with 50% cell viability at 5 nM, while GBM#41 (EGFRvIII) showed no significant change in cell growth with only a 20% decrease in viability at a concentration of 500 nM (Figure 1B). Uridine supplementation diminished the efficacy of BAY2402234 on GBM#35 cells (Figure 1C), indicating the reliance of GBM#35 on DHODH dependent nucleotide synthesis pathways. Treatment with 5 nM and 10 nM BAY2402234 resulted in distinct expression profiles of p53 induction and cleaved caspase 3 in EGFRWT cells (Figure 1D,E). BAY2402234 did not alter the basal levels of AKT and MAPK in these cells (Figure 1D). While BAY2402234 treatment led to a dose-dependent enhancement of caspase 3 cleavage in GBM#35, the treatment of GBM#41 cells (EGFRvIII) did not elicit changes in caspase 3 cleavage (Figure 1E), suggesting that the inhibition of DHODH induced apoptotic cell death in EGFRWT but not in EGFRvIII-expressing cells.

### 3.2. Treatment with the DHODH Inhibitor BAY2402234 Induces Triglyceride Accumulation at the Expense of Membrane Lipids in GBM Cells Harboring the Wild-Type EGFR Gene

**Figure 2:**
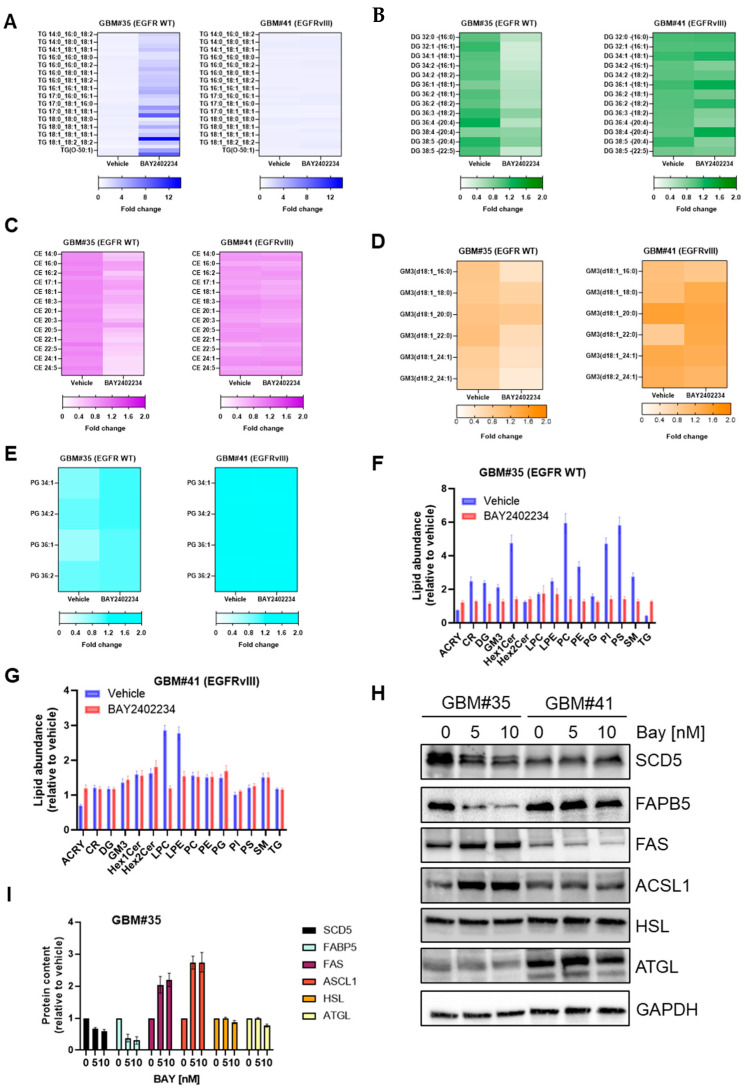
Lipidomics analysis of GBM#35 and GBM#41 cell lines treated with DHODH inhibitor BAY2402234 (Bay). A fold change in (**A**) triglyceride (TG), (**B**) diglyceride (DG), (**C**) ceramide (CE), (**D**) ganglioside 3 (GM3), (**E**) phosphatidylglycerol (PG) and (**F**,**G**) other lipids in GBM#35 and GBM#41 treated with Bay 10 nM for 48 h. (**H**,**I**) Steady-state level of fatty acid and lipid metabolism regulating proteins in GBM#35 and GBM#41 cells treated with indicated concentration of Bay. For quantitation in I, protein abundance in vehicle-treated cells is set to 1.

We aimed to investigate the metabolic interactions of BAY2402234 therapy at the cellular level. Lipids play pivotal roles in cancer development and progression, serving as building blocks for cell membranes, energy storage, and signaling molecules [17,20,35]. We assessed the impact of BAY2402234 on lipid abundance in both EGFR wild-type and mutant GBM cell lines. Initially, we examined triglycerides stored in lipid droplets and metabolized into free fatty acids during cellular stress. Treatment with 10 nM of BAY2402234 for 48 h increased triglyceride accumulation approximately 12-fold in GBM#35 cells, whereas triglyceride content in GBM#41 cells remained unaffected by the drug (Figure 2A). 

We also analyzed other lipid classes including sphingolipids and glycerophospholipids, which are involved in signaling pathways that regulate cell proliferation, apoptosis, migration, and angiogenesis and thus contribute to tumor progression [16,17,18,19,20,21]. Treatment with BAY2402234 did not alter diglyceride and ceramide lipid levels in GBM#41 cells, but it decreased the abundance of diglyceride (DG) and cholesteryl esters (CE) approximately 2-fold in GBM#35 cells (Figure 2B,C). Similarly, BAY2402234 treatment reduced structural lipids like gangliosides (GM) and phosphotidylglycerol (PG) in GBM#35 cells while showing no significant impact on the abundance of GM and PG in GBM#41 cells (Figure 2D–G, Table S1). However, we observed a significant change in the abundance of lyso-phosphotidylcholine (LPC) and lyso-phosphotidylethanolamine (LPE) in BAY2402234-treated GBM#41 cells (Figure 2G, Table S1). BAY2402234-treated GBM#35 cells also showed a change in the abundances of some of fatty acid and lipid metabolism-regulating proteins (Figure 2H,I). BAY2402234 treatment reduced the abundance of enzymes involved in the biosynthesis of unsaturated fatty acids like stearoyl-CoA desaturase 5 (SCD5) and intercellular lipid transporter fatty acid-binding protein 5 (FAPB5) in GBM#35 while showing no significant impact on GBM#41 cells. The abundance of fatty acid synthase (FAS) and acyl-CoA synthetase long-chain 1 (ACSL1) increased in BAY2402234-treated GBM#35 cells (Figure 2H,I), while the treatment of GBM#41 cells (EGFRvIII) did not elicit changes in FAS and ACSL1 expression levels (Figure 2H). Lipolytic enzyme hormone-sensitive lipase (HSL) and adipose triglyceride lipase (ATGL) that involved in the breakdown of stored triglycerides did not show a change in expression level in both BAY2402234-treated GBM#35 and GBM#41 cells (Figure 2H). These findings suggest that the inhibition of pyrimidine metabolism enhances neutral lipid storage, which is likely at the expense of lipid subclasses mediating the oncogenic signaling pathways and membrane structure in GBM cells harboring the wild-type EGFR gene.

### 3.3. BAY2402234 Treatment Exerts Differential Regulation on Fatty Acid Metabolism and TCA Cycle Metabolic Flux in EGFR WT Cells

**Figure 3:**
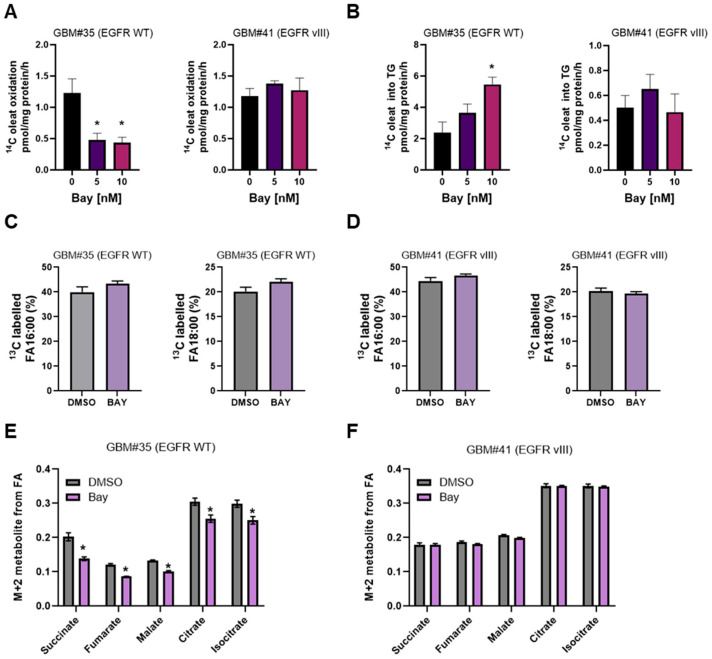
Carbon tracer-based analyses of GBM cell fatty acid metabolism treated with DHODH inhibitor BAY2402234 (Bay). (**A**,**B**) ^14^C oleate utilization of GBM cells treated with Bay 10 nM for 48 h. (**C**,**D**) ^13^C fatty acid mix uptake of GBM cells treated with Bay 10 nM for 48 h (n = 4, * *p* < 0.05). (**E**,**F**) ^13^C fatty acid mix carbon flux into TCA cycle in GBM cells treated with Bay 10 nM for 48 h (n = 4, * *p* < 0.05).

Given the profound effects of BAY2402234 on remodeling of the lipide in EGFR WT cells, we next investigated the effects of DHODH inhibition on fatty acid metabolism and TCA cycle flux in cells. We traced the fate of ^14^C-oleate for 4 h in GBM cells treated with BAY2402234. BAY2402234-treated EGFRWT cells exhibited a 50% decrease in fatty acid oxidation (Figure 3A, left panel) alongside a BAY2402234 dose-dependent increase in the esterification of ^14^C oleic acid into triglyceride (Figure 3B, left panel). This is consistent with the 12-fold increase in TG content in these cells after prolonged BAY2402234 administration (Figure 2A). Conversely, we did not observe changes in FA oxidation and storage in GBM#41 cells treated with various concentrations of BAY2402234 (Figure 3A,B, right panel). 

We next conducted post hoc analyses of our earlier study investigating fatty acid handling in BAY2402234-treated GBM cells using ^13^C uniformly labeled mixed fatty acids containing myristic (0.2%), palmitoleic (9.4%), palmitic (38.9%), margaric (0.3%), linoleic (10.7%), oleic (26.9%), elaidic (1.6%), and stearic (1.6%) acids via GC-MS. There were no notable differences in the enrichment of ^13^C mixed fatty acids in EGFRWT or EGFRvIII cells under basal or BAY2402234-treated conditions, indicating similar levels of fatty acid uptake (Figure 3C,D). In contrast, BAY2402234 treatment attenuated fractional enrichment in the M+2 isotopologue of succinate, fumarate, malate, citrate, and isocitrate in EGFRWT cells, indicating reduced TCA cycle activity (Figure 3E). Conversely, we did not observe changes in TCA cycle flux in EGFRvIII cells treated with BAY2402234 (Figure 3F). These findings concur with the ^14^C-fatty acid oxidation experiments (Figure 3A) and unveil a novel mechanism by which EGFR wild-type but not mutant gene-harboring GBM cells modulate FA oxidation in response to BAY2402234 treatment.

## 4. Discussion

The interplay between metabolic reprogramming and genetic mutations in response to drug treatment is crucial for acquired drug resistance [35,36]. In this study, we investigated whether mutations in the EGFR gene in glioblastoma lead to lipid metabolic-dependent phenotypes in response to the blood–brain barrier penetrant drug BAY2402234. Our findings demonstrate that EGFR wild-type (WT) GBM cells exhibit sensitivity to BAY2402234 treatment and undergo changes in lipid droplet metabolite abundance, highlighting the importance lipid utilization in drug resistance. In contrast, EGFRvIII glioblastoma cells display insensitivity to BAY2402234 and show minimal lipid metabolic reprogramming. 

Temozolomide (TMZ) is a brain penetrant therapeutic agent that has been part of the standard of care treatment for glioblastoma since 2005 [4,5]. However, despite its widespread use, progression typically occurs within months after initiating these treatments, the adaptability of glioblastoma to TMZ remains poorly understood [4,5,9], and no new medical therapies have been approved for adult patients with glioblastoma in the last two decades [37,38,39,40]. 

Recently, the dihydroorotate dehydrogenase (DHODH) inhibitor BAY2402234 displayed efficacy in different brain cancer animal models. DHODH is localized on the inner mitochondrial membrane and crucial for de novo pyrimidine nucleotide production, which is initiated with the generation of uridine monophosphate (UMP) [41,42]. Although DHODH is ubiquitously expressed in every human organ and has less than 4% mutation and alternation in cancer [43,44], malignant cells appear to be more metabolically dependent on de novo pyrimidine production. Therefore, this dependency forms the potential basis of a therapeutic window to selectively target this vulnerability in malignant cells. 

Lipid droplets, also known as lipid depots, are organelles that compartmentalize neutral lipids within a hydrophobic matrix covered by proteins embedded in a phospholipid monolayer [45,46]. While they play a crucial role in lipid homeostasis, recent attention has shifted toward more mechanistic inquiries regarding lipid droplet function in sequestering biomolecules and providing protection from cytotoxic molecules [35,47,48]. Our data demonstrate that EGFR-driven GBM cells downregulate fatty acid oxidation and reduce the abundance of structural lipids that presumably supports the biogenesis of stored lipid triglycerides (Figure 2 and Figure 3). An advantageous aspect of increased lipid droplet abundance could be to reduce lipotoxicity and store energy, which is in high demand during tumor cell migration. Previous studies have shown that KRAS-driven cancers rely less on fatty acid oxidation for steady-state proliferation, shifting toward lipolysis and the oxidation of stored lipids during invasion and metastasis [49]. Conversely, the abundance of potential cytotoxic lipids such as acylcarnitines and ceramide may uncouple the mitochondrial membrane potential and disrupt mitochondrial function [50,51]. Therefore, it is plausible that EGFR wild-type cells undergo a shift toward lipid droplet accumulation as a protective mechanism against oxidative damage or as a reserve of energy for invasion and metastasis. These aspects warrant further investigation that shed light on an unrecognized aspect of the cellular adaptive response to starvation mediated by lipid droplets.

In summary, our study elucidates that the metabolic switch in response to drug treatment is dependent on the functional genomic features of GBM cells. This insight is pivotal in understanding metabolic-based drug resistance in the context of patient-specific genetic alterations. These findings hold significant implications for further clinical research, paving the way for the development of novel multi-targeted inhibitors.

## Data Availability

The original contributions presented in the study are included in the article and Supplementary Material.

## Supplementary Materials

The following supporting information can be downloaded at: https://www.mdpi.com/article/10.3390/metabo14080413/s1, Table S1: The lipidomic profile of GBM#35 and GBM#41 cells treated with DHODH inhibitor BAY2402234.
